# Association of Lower Plasma Homoarginine Concentrations with Greater Risk of All-Cause Mortality in the Community: The Framingham Offspring Study

**DOI:** 10.3390/jcm9062016

**Published:** 2020-06-26

**Authors:** Edzard Schwedhelm, Rebecca J. Song, Ramachandran S. Vasan, Edwin R. van den Heuvel, Juliane Hannemann, Vanessa Xanthakis, Rainer Böger

**Affiliations:** 1 Institute of Clinical Pharmacology and Toxicology, University Medical Center Hamburg-Eppendorf, Hamburg, and DZHK Partner Site Hamburg/Kiel/Lübeck, 20246 Hamburg, Germany; j.hannemann@uke.de (J.H.); boeger@uke.de (R.B.); 2 Department of Epidemiology, Boston University School of Public Health, Boston, MA 02118, USA; rsong@bu.edu (R.J.S.); vasan@bu.edu (R.S.V.); 3 Section of Preventive Medicine and Epidemiology, Boston University School of Medicine, Boston, MA 02118, USA; evandenh@bu.edu (E.R.v.d.H.); vanessax@bu.edu (V.X.); 4 Framingham Heart Study, Framingham, MA 01702, USA; 5 Computing and Data Sciences Institute, Boston University, Boston, MA 02215, USA; 6 Department of Biostatistics, Boston University School of Public Health, Boston, MA 02118, USA

**Keywords:** cardiovascular disease, homoarginine, nitric oxide, risk factors

## Abstract

Lower circulating homoarginine concentrations have been associated with morbidity and mortality in patients with established cardiovascular disease (CVD). We assayed plasma homoarginine concentrations in 3331 Framingham Offspring Study participants attending examination cycle six (mean age 58.6 years, 53% women). We evaluated correlates of plasma homoarginine and related homoarginine to incident CVD and death. We also classified participants as having higher (upper quartile) versus lower (lower three quartiles) homoarginine and previously assayed asymmetric dimethylarginine (ADMA) concentrations, and created cross-classification groups. We observed 630 incident CVD events and 940 deaths during a median follow-up of 18 years. In multivariable regression analysis, homoarginine was associated positively with male sex, body mass index, anti-hypertensive medication use and systolic blood pressure, but inversely with age and smoking. Higher homoarginine levels were associated with a lower mortality risk (hazard ratio (HR) per SD increment, 0.83, 95% CI: 0.74–0.93) adjusting for standard CVD risk factors, and ADMA. Among the cross-classification groups, participants with higher homoarginine and lower ADMA had a lower mortality risk (HR, 0.81, 95% CI: 0.67–0.98) compared to those with low levels of both. Further studies are needed to dissect the mechanisms of the association of homoarginine and mortality over decades in the community.

## 1. Introduction

Clinical evidence suggests that lower circulating homoarginine concentrations are a risk factor for cardiovascular disease, and all-cause mortality [1,2]. Patients with prevalent ischemic stroke, ischemic heart disease, and congestive heart failure have low blood homoarginine concentrations [3,4,5]. Consistent with the same line of evidence, homoarginine supplementation in mouse models of stroke and heart failure has been shown to improve neurological outcomes and preserve cardiac function, respectively [3,6].

In vitro and in vivo evidence shows that the amidinotransferase L-arginine: glycine amidinotransferase (AGAT, EC 2.1.4.1) transfers the guanidine group of L-arginine to L-lysine, catalyzing the biosynthesis of L-homoarginine in vertebrates [7,8,9]. Furthermore, the AGAT gene locus is linked to interindividual variation in plasma homoarginine in humans [3,10]. Mean circulating concentrations of homoarginine are about 2 µM in humans [11,12]. Homoarginine is a constituent of the human diet [13,14] and it can be supplemented orally in healthy human subjects without causing harm [15,16]. Due to its structural similarity with L-arginine, homoarginine influences L-arginine metabolism. It competitively inhibits arginase [17], serves as substrate for nitric oxide synthase (NOS), and allosterically inhibits alkaline phosphatase [1]. In contrast, asymmetric (ADMA) and symmetric dimethylarginine (SDMA) are direct and indirect inhibitors of NOS, respectively [18]. Several reports have underscored the detrimental effect of ADMA and the salutary effect of homoarginine, respectively, on vascular function and biology [1,18]. Plasma homoarginine concentrations correlated positively with brachial artery diameter and flow-mediated dilatation in pregnant women during the second and third trimester [19]. Moreover, homoarginine was noted to inhibit the aggregation of human platelets stimulated with collagen in a manner similar to L-arginine [20].

In previous reports, higher plasma ADMA concentrations have been associated with a higher risk of all-cause mortality in the Framingham Offspring cohort [21]. Limited evidence suggests that homoarginine and ADMA may be associated with cardiovascular disease (CVD) risk and mortality in a reciprocal manner in population-based cohorts. Accordingly, we hypothesized that higher homoarginine and lower ADMA concentrations are associated with a lower CVD risk and mortality in the community. We tested this hypothesis using data from the Framingham Offspring Study participants who attended a routine examination in which both analytes were assayed.

## 2. Experimental Section

### 2.1. Study Design

The design and selection criteria of the Framingham Offspring Study have been described previously [22]. The 3532 participants who attended the 6th examination cycle (1995 through 1998) were eligible for the present investigation. We excluded 201 attendees for the following reasons: serum creatinine above 2.0 mg/dL (177 μmol/L) suggesting some degree of renal impairment (*n* = 21), and non-available homoarginine levels (*n* = 180), resulting in a sample size of *n* = 3331 (Sample 1). After excluding participants with prevalent CVD (*n* = 366; which includes prevalent peripheral artery disease (PAD) *n* = 60; ischemic stroke or transient ischemic attack (TIA) *n* = 53; coronary heart disease (CHD) *n* = 242; and congestive heart failure (HF) *n* = 11), Sample 2 included 2965 participants. The study protocol was approved by the Institutional Review Board of the Boston University Medical Center and the Ethics Committee of the Hamburg Board of Physicians. All participants provided written informed consent.

### 2.2. Biomarkers

We used the following biomarkers for this investigation: homoarginine (primary exposure), ADMA, C-reactive protein (CRP), brain natriuretic peptide (BNP), growth/differentiation factor 15 (GDF15), and high sensitivity troponin I. Laboratory assessment of all biomarkers was conducted on samples drawn from participants during their routine examination at the Framingham Offspring 6th examination cycle. Briefly, phlebotomy was performed (typically between 8 a.m. and 9 a.m.) on fasting participants who were supine for approximately 5 to 10 min. Blood was immediately centrifuged, and the plasma/ serum separated and stored at −80 °C until assayed. Plasma samples for the determination of homoarginine measurements were processed using a validated liquid chromatography-tandem mass spectrometric (LC–MS/MS) assay [12]. Briefly, 25 μL aliquots of plasma were spiked with an internal standard, i.e., stable isotope-labelled homoarginine. By adding 100 µL methanol, proteins were precipitated. After filtrating the samples through a 0.22 μm hydrophilic membrane (Multiscreen HTS™, Millipore, Molsheim, France), analytes were derivatized to their butylester derivatives with butanolic 1 N HCl, and analyzed by a LC-MS/MS (Varian 1200 MS, Agilent Technologies, Santa Clara, USA). The measurements of ADMA, CRP, BNP, GDF15, and troponin I have been previously described [21,23].

### 2.3. Outcomes

The outcomes of interest were the incidence of a first CVD event and all-cause mortality during follow-up from the 6th exam cycle. Major CVD events included fatal or nonfatal CHD (myocardial infarction, coronary insufficiency, and angina pectoris), stroke, TIA, intermittent claudication, PAD, or HF. Criteria for these events have been described elsewhere [22]. A committee of three experienced physicians reviewed and adjudicated all suspected events by examining medical records from hospitalizations, physician office visits, and Heart Study visits; these investigators were blinded to homoarginine plasma concentrations.

### 2.4. Statistical Analysis

We evaluated the clinical correlates of homoarginine using multivariable linear regression with backward selection, with a selection cut-off of *p* < 0.10 (dependent variable: homoarginine; candidate independent variables included age, sex, smoking status, diabetes, systolic blood pressure (SBP), diastolic blood pressure (DBP), body mass index (BMI), ratio of total/high-density lipoprotein (HDL) cholesterol, anti-hypertensive medication, prevalent CVD, ADMA, and estimated glomerular filtration rate (eGFR)). The Chronic Kidney Disease-Epidemiology Collaboration (CKD-EPI) equation was used to calculate eGFR. We presented Kaplan–Meier curves for the quartiles of homoarginine. We visualized the crude mortality rates in a 3D plot to show patterns of risk by quartiles of homoarginine and ADMA in our sample. We used a Cox proportional hazards regression model to relate homoarginine (standardized independent variable) to the risk of all-cause mortality (*n* = 3331, Sample 1).

First, we performed a minimally-adjusted model including age and sex, and then we evaluated a multivariable-adjusted model that included age, sex, SBP, BMI, current smoking, use of anti-hypertensive medications, diabetes, total cholesterol/HDL ratio, prevalent CVD, and ADMA. The proportional hazards (PH) assumption was violated for models assessing all-cause mortality (log (follow-up time) * homoarginine interaction *p*-value = 0.001). Upon assessment of the weighted Schoenfeld residuals plotted against time to death, the trend of the residuals did not seem to show an extreme violation. Nevertheless, we conducted a weighted Cox proportional hazards model that estimates a weighted hazard ratio from different time points during follow-up in the presence of non-proportional hazards [24].

Then, we assessed the relations between homoarginine (standardized independent variable) and incident CVD using a Cox proportional hazards model, after excluding participants with prevalent CVD (*n* = 2965, Sample 2). We performed a minimally-adjusted and a multivariable-adjusted model using the same covariates as detailed above for the all-cause mortality outcome. The proportional hazards assumption was met for models assessing incident CVD. In sensitivity analyses, we further included eGFR, blood CRP, BNP, GDF15, or troponin I concentrations as covariates in models evaluating the risk of all-cause mortality. As ADMA was positively associated with all-cause mortality in our previous analysis [21], we assessed the possible interaction between homoarginine and ADMA in models evaluating the risk of all-cause mortality. We created cross-classification groups of higher versus lower homoarginine and ADMA as follows: we first created quartiles of homoarginine and ADMA in our sample. Participants with homoarginine or ADMA levels in the highest quartile were considered “higher”, and participants in the lower three quartiles were considered “lower”, resulting in four groups as follows: high homoarginine, high ADMA; high homoarginine, low ADMA; low homoarginine, high ADMA; and low homoarginine, low ADMA (referent group). Lastly, we performed a Cox proportional hazards regression model with the cross-classification groups of homoarginine and ADMA as the main exposure and risk of total mortality as the outcome of interest.

All analyses were performed with the use of SAS software (version 9.4). A two-sided *p* value of less than 0.05 was considered to indicate statistical significance.

## 3. Results

The baseline characteristics of the participants are shown in Table 1. The mean ± standard deviation concentration of homoarginine was 1.66 ± 0.73 µmol/L with a median (25th and 75th percentile) of 1.56 (1.14, 2.07) µmol/L.

### 3.1. Correlates of Circulating Homoarginine

In multivariable regression analysis using backward selection with *p* < 0.10, plasma homoarginine concentration was positively associated with male sex, BMI, anti-hypertensive medication use and systolic blood pressure, but was inversely related to age and current smoking status (Table 2). Clinical variables explained about 7.6% of the inter-individual variation in circulating homoarginine concentrations.

### 3.2. Relations of Homoarginine and All-Cause Mortality

**Figure 1:**
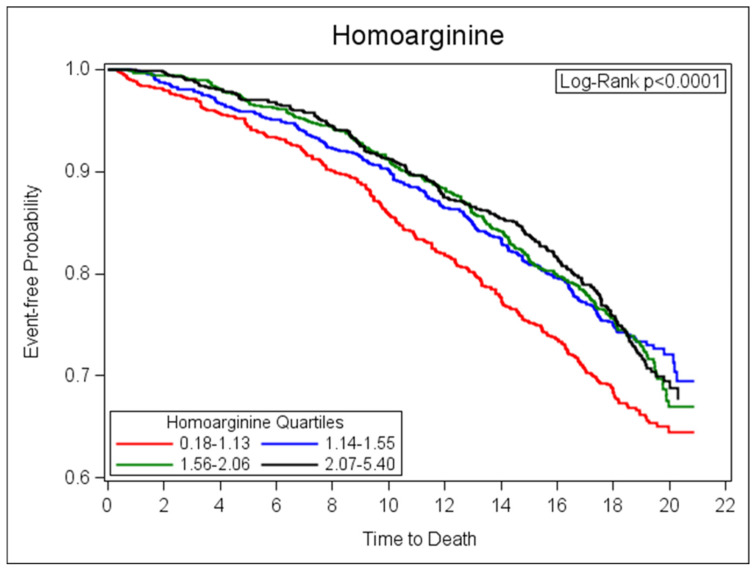
Kaplan–Meier curves for quartiles of homoarginine and event-free probability of survival (years).

Among the 3331 participants, we observed 940 deaths over a median follow-up of 18.2 (Q1:16.2, Q3:19.3) years. Figure 1 shows Kaplan–Meier survival curves for quartiles of homoarginine. The mortality rate was higher in the quartile (Q) with the lowest homoarginine plasma concentration (Q1) compared to Q2–Q4. The median (25th percentile, 75th percentile) of homoarginine in Q1 was 0.90 (0.72, 1.03) µmol/L, and in Q4 was 2.48 (2.24, 2.85). Higher homoarginine values were associated with a lower mortality risk in the multivariable-adjusted weighted Cox proportional hazards model (Table 3). In sensitivity analyses, higher homoarginine values were associated with lower mortality risk in a multivariable model additionally adjusting for eGFR, and blood CRP, BNP, GDF15, or troponin I concentrations (Supplementary Table).

**Figure 2:**
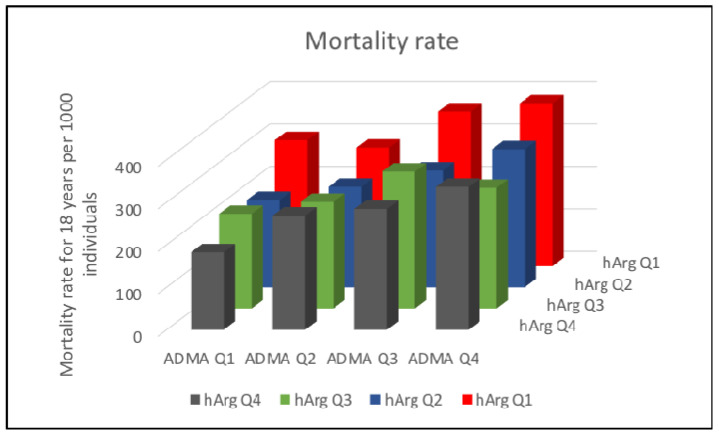
Crude mortality rates for 18 years of follow-up and quartiles of homoarginine (hArg) and asymmetric dimethylarginine (ADMA). The depicted quartiles (Q) are as follows: hArg Q1: 0.18–1.13 µmol/L, hArg Q2: 1.14–1.55, hArg Q3: 1.56–2.06 µmol/L, hArg Q4: 2.07–5.40 µmol/L, ADMA Q1: 0.14–0.46 µmol/L, ADMA Q2: 0.46–0.53 µmol/L, ADMA Q3: 0.53–0.61 µmol/L, ADMA Q4:0.62–1.37 µmol/L.

In analyses using the cross-classification groups, we observed lower mortality risk among participants with higher (highest quartile) homoarginine values and lower ADMA (lower three quartiles combined) compared to those with lower homoarginine and lower ADMA [HR, 0.81, 95% CI: 0.67–0.98] (Table 4). We observed decreasing mortality rates for increasing quartiles of homoarginine and increasing mortality rates for increasing quartiles of ADMA (Figure 2).

### 3.3. Relations of Homoarginine and Incident CVD

There were 630 incident CVD events among 2965 individuals during follow-up. Incident CVD events comprised 268 CHD events (including CHD-related deaths), 187 stroke/TIA events (including stroke-related deaths), 125 HF events, 45 PAD events, and 5 other CVD-related deaths. Plasma homoarginine was not associated with incident CVD in a multivariable Cox proportional hazards model adjusting for established risk factors and ADMA (Table 3).

## 4. Discussion

Our observations from this investigation are two-fold: First, lower circulating homoarginine levels are associated with a higher mortality risk during a follow-up period of more than 18 years, adjusting for known CVD risk factors; second, ADMA and homoarginine are inter-related with regards to mortality risk such that higher homoarginine and lower ADMA levels are associated with a lower risk of death.

### 4.1. Homoarginine in the Community

In our investigation, median homoarginine plasma concentration was relatively low (1.56 µmol/L) as compared with the 1.88 µmol/L that was reported previously for healthy humans [25]. Plasma reference intervals determined from these individuals decline with age and are lower in women when compared with men [25]. For older individuals (mean age 70 years) participating in the Hoorn Study, even lower homoarginine plasma concentrations were reported, i.e., 1.46 µmol/L [26]. Recent results from genome-wide association (GWA) studies identified a strong link between plasma homoarginine and the enzyme AGAT [3,10]. Likewise, mice with genetic deletion of AGAT exhibit low homoarginine concentrations in plasma and tissues [3,27]. In addition to the biosynthesis of homoarginine, AGAT is also the rate-limiting enzyme in creatine synthesis [11]. Creatine represents an energy buffer in skeletal muscle, brain, heart and several other high energy demand tissues. In our cross-sectional evaluation of correlates of homoarginine, we observed positive associations of homoarginine with male sex and BMI which might be attributable to higher skeletal muscle mass and, thus, creatine levels.

Results from several previous studies in patients with prevalent CVD showed an association between low homoarginine plasma concentrations and a higher risk of CVD (i.e., sudden cardiac death, heart failure, and myocardial infarction), cerebrovascular outcomes, and mortality (both all-cause and CVD-related) [1,2,3,4,5]. In the present investigation, we confirmed the association of homoarginine with all-cause mortality in a large community-based sample that included few people with prevalent CVD, and we extended our analyses to also include CRP, BNP, GDF15, eGFR, and troponin I as covariates. Furthermore, our participants were followed for a much longer period (median 18 years) when compared to other studies, such as the Dallas Heart Study (9.4 years [28]) and the Hoorn Study (7.8 years [26]). Notably, we did not observe an association between plasma homoarginine concentration and incident CVD. This observation is in line with a previously reported association between ADMA and all-cause mortality but not with CVD in same study cohort [21]. It is of note that a combination of high homoarginine and low ADMA was associated with the lowest mortality rate, and mortality risk gradually increased with both decreasing homoarginine and increasing ADMA concentrations, further suggesting a role of both biomarkers in mediating mortality risk.

The exact molecular function(s) of homoarginine that may underlie its association with mortality remain unclear. On one hand, experimental evidence supports a role for homoarginine as a low-affinity substrate for NO synthesis. However, it is not clear whether, under physiological conditions, homoarginine acts as a substrate enhancing NO generation or rather decreases NO production by competing with L-arginine [17], the main NOS substrate. On the other hand, homoarginine is generated by the activity of AGAT, an enzyme that is also pivotal for the synthesis of creatine, an important reservoir for high energy phosphate groups in muscle [11,29]. We have shown previously that mice lacking AGAT have very low homoarginine concentrations and are susceptible to develop large strokes upon experimental carotid artery ligation [3]. This effect remained unchanged when creatine was supplemented, but it was completely reversible when homoarginine was supplemented, suggesting that homoarginine may have a creatine-independent effect on cerebrovascular ischemic sequelae. In the same line of evidence, AGAT-deficient mice show hemodynamic impairment with reduced cardiac inotropy, lusitropy, and contractile reserve; all of these cardiac phenotypes were rescued by homoarginine supplementation [27]. In contrast to ADMA, homoarginine can be supplemented in humans without any harm observed so far [15,16]. Thus, further studies are needed to evaluate any potential beneficial effect of homoarginine supplementation on morbidity and mortality in individuals with low plasma homoarginine concentration.

### 4.2. Strengths and Limitations

The strengths of our study are its prospective design, the longitudinal surveillance over more than 18 years for occurrence of CVD and death, and the availability of data on other biomarkers (including ADMA) among Framingham Offspring participants. However, some of the limitations of our approach also warrant acknowledgment. Establishing that lower homoarginine levels are a risk factor for all-cause mortality prospectively requires additional mechanistic investigations, randomized controlled trials, and study of cause-specific mortality patterns. In addition, the majority of our sample is white of European descent, limiting the generalizability to other ethnicities.

## 5. Conclusions

In our large community-based sample, lower plasma homoarginine concentrations were associated with higher mortality risk during follow-up. Additional studies are warranted to replicate these findings. If replicated, randomized clinical trials will be needed to elucidate the suitability of dietary supplementation with homoarginine to lower the risk of morbidity and mortality.

## Figures and Tables

**Table 1:** Baseline characteristics of study sample.

Characteristic ^1^	Men (*N* = 1552)	Women (*N* = 1779)
Age (yr)	59 ± 10	59 ± 10
Current cigarette smoking (%)	14	16
Total cholesterol (mg/dL)	199 ± 41	212 ± 39
HDL cholesterol (mg/dL)	43 ± 12	58 ± 16
Total/HDL Ratio	4.9 ± 2.0	4.0 ± 1.4
Body Mass Index (kg×m^2^)	28.5 ± 4.4	27.4 ± 5.7
Systolic Blood Pressure (mmHg)	130 ± 17	127 ± 20
Diastolic Blood Pressure (mmHg)	77.2 ± 9.5	73.8 ± 9.2
Hypertension (%)	45	38
Use of antihypertensive agents (%)	31	25
Blood glucose (mg/dL)	107 ± 28	100 ± 26
Diabetes mellitus (%)	12	8
Serum creatinine (mg/dL)	1.24 ± 0.19	1.07 ± 0.18
eGFR (mL/min/1.73 m^2^)	86 ± 18	85 ± 19
Prevalent CVD (%)	15	7
Homoarginine (µmol/L)	1.73 (1.33, 2.23)	1.38 (1.01, 1.88)
ADMA (µmol/L)	0.54 (0.47, 0.62)	0.53 (0.46, 0.61)
CRP (mg/dL)	1.8 (0.9, 3.8)	2.4 (1.0, 5.8)
GDF15 (ng/L)	1058 (822, 1406)	1021 (811, 1305)
BNP (pg/mL)	6.6 (4.0, 16.8)	10.0 (4.0, 20.2)
Troponin I (pg/mL)	1.63 (1.05, 2.65)	1.15 (0.78, 1.91)

^1^ Values are means ± SD or median (25th and 75th percentile) unless otherwise indicated. ADMA, asymmetric dimethylarginine; BNP, b-type natriuretic peptide; CRP, C-reactive protein; eGFR, glomerular filtration rate; GDF15, growth/differentiation factor 15; HDL, high-density lipoprotein. To convert values for cholesterol to millimoles per liter, multiply by 0.02586. To convert values for creatinine to millimoles per liter, multiply by 88.4. To convert values for glucose to mmoles per liter, multiply by 0.0555.

**Table 2:** Cross-sectional correlates of plasma homoarginine.

Variable ^1^	Unit of Increase	Regression Coefficient (SE)	*p* Value
Sex	men vs. women	0.293 (0.025)	<0.001
Smoking	current vs. not current	−0.195 (0.035)	<0.001
Age	10 years	−0.051 (0.016)	0.002
BMI	1 kg×m^2^	0.013 (0.002)	<0.001
Anti-hypertensive medication use	yes vs. no	0.069 (0.030)	0.019
Systolic blood pressure	10 mm Hg	0.017 (0.007)	0.022
eGFR	1 mL/min	0.001 (0.001)	0.063

^1^ Independent variables reported are those that remained in the model after stepwise backward elimination analysis and were statistically significant in the final model (p<0.10). Candidate correlates were chosen on the basis of significant univariate associations and pathophysiological mechanisms. R2 of the final model was 0.076 for homoarginine. SD denotes standard derivation. BMI, body mass index; eGFR, estimated glomerular filtration rate.

**Table 3:** Association of plasma homoarginine concentration with risk of cardiovascular disease (CVD) and all-cause mortality.

Outcome	Age- and Sex-Adjusted HR (95% CI) ^2^	*p* Value	Multivariable-Adjusted ^1^ HR (95% CI)	*p* Value
All-cause mortality	0.81 (0.72–0.91)	<0.001	0.83 (0.74–0.93)	0.002
Incident CVD	1.12 (1.00–1.25)	0.041	1.06 (0.95–1.19)	0.291

^1^ Adjusted for age, sex, systolic blood pressure, body mass index, current smoking, use of anti-hypertensive medications, diabetes, total cholesterol/HDL cholesterol ratio, prevalent CVD, and ADMA. Prevalent CVD was excluded in models assessing incident CVD. ^2^ Hazard ratios (HR) are per 1-SD increase in homoarginine.

**Table 4:** Association of cross-classification groups of plasma homoarginine and ADMA concentrations with risk of all-cause mortality.

Cross-Classification Group	Multivariable-Adjusted ^1^ HR (95% CI)	*p* Value
Low Homoarginine and Low ADMA	1.00 (ref)	
Low Homoarginine and High ADMA	0.97 (0.82, 1.15)	0.722
High Homoarginine and High ADMA	0.99 (0.77, 1.28)	0.958
High Homoarginine and Low ADMA	0.81 (0.67, 0.98)	0.028

^1^ Adjusted for age, sex, systolic blood pressure, body mass index, current smoking, use of anti-hypertensive medications, diabetes, total cholesterol/HDL cholesterol ratio, prevalent CVD.

## Supplementary Materials

The following are available online at https://www.mdpi.com/2077-0383/9/6/2016/s1 Table S1: Association of plasma homoarginine with risk of all-cause mortality further adjusting for eGFR, CRP, BNP, GDF15 or troponin I.
