# Supplementary material for: Association of Lower Plasma Homoarginine Concentrations with Greater Risk of All-Cause Mortality in the Community: The Framingham Offspring Study
Source: J Clin Med. 2020 Jun 26;9(6):2016. doi: 10.3390/jcm9062016 (PMC7356383; doi:10.3390/jcm9062016)
Supplement: Supplementary file 1 [file jcm-09-02016-s001.pdf]

Supplementary Table 1. Association of plasma homoarginine with risk of all-cause mortality further adjusting for eGFR, CRP, BNP, GDF15 or troponin I.

Variable *	Fully Adjusted HR 95 % CI **	*P* value
Homoarginine	0.83 (0.74–0.93)	0.002
+eGFR	0.83 (0.74–0.94)	0.002
+CRP	0.78 (0.67–0.91)	0.002
+BNP	0.84 (0.75–0.95)	0.003
+GDF15	0.87 (0.78–0.97)	0.013
+Troponin I	0.83 (0.74–0.93)	0.002

* We adjusted models for age, sex, systolic blood pressure, body mass index, current smoking, use of anti-hypertensive medications, diabetes, total cholesterol/HDL cholesterol ratio, prevalent CVD, ADMA, homoarginine and additionally for eGFR, CRP, BNP, GDF15 or troponin I, respectively. Biomarkers were standardized to one standard deviation increase. BNP, b-type natriuretic peptide; CRP, C-reactive protein; eGFR, glomerular filtration rate; GDF15, growth/differentiation factor 15. ** HRs are per 1-SD increase in homoarginine.
